# Supplementary figures and images for: Profile of Bioactive Components of Cocoa (Theobroma cacao L.) By-Products from Ecuador and Evaluation of Their Antioxidant Activity
Source: Foods. 2023 Jul 3;12(13):2583. doi: 10.3390/foods12132583 (PMC10341204; doi:10.3390/foods12132583)

Figure S1. Standards of phenolic compounds

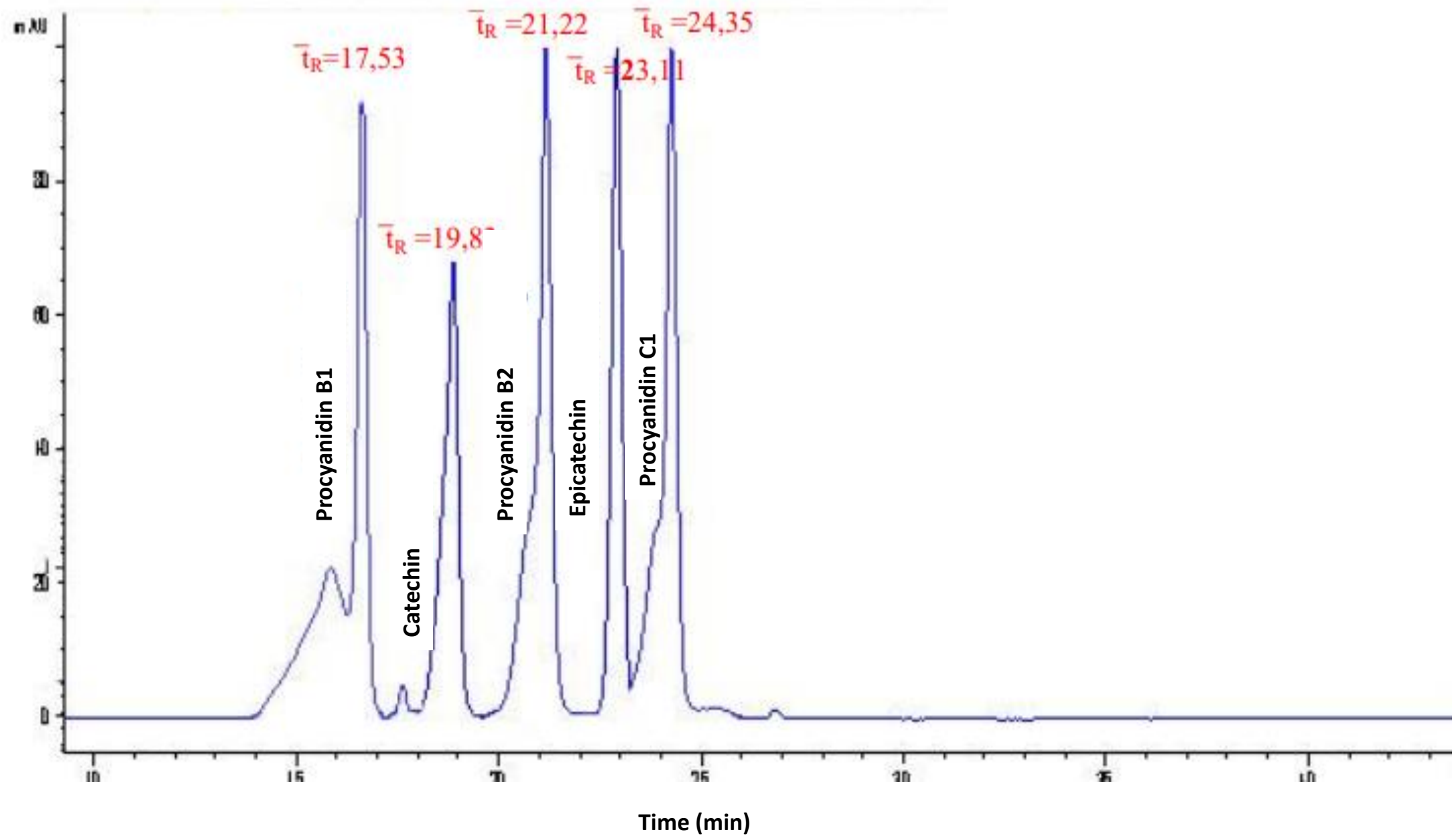

Figure S2. Standards of methylxanthines

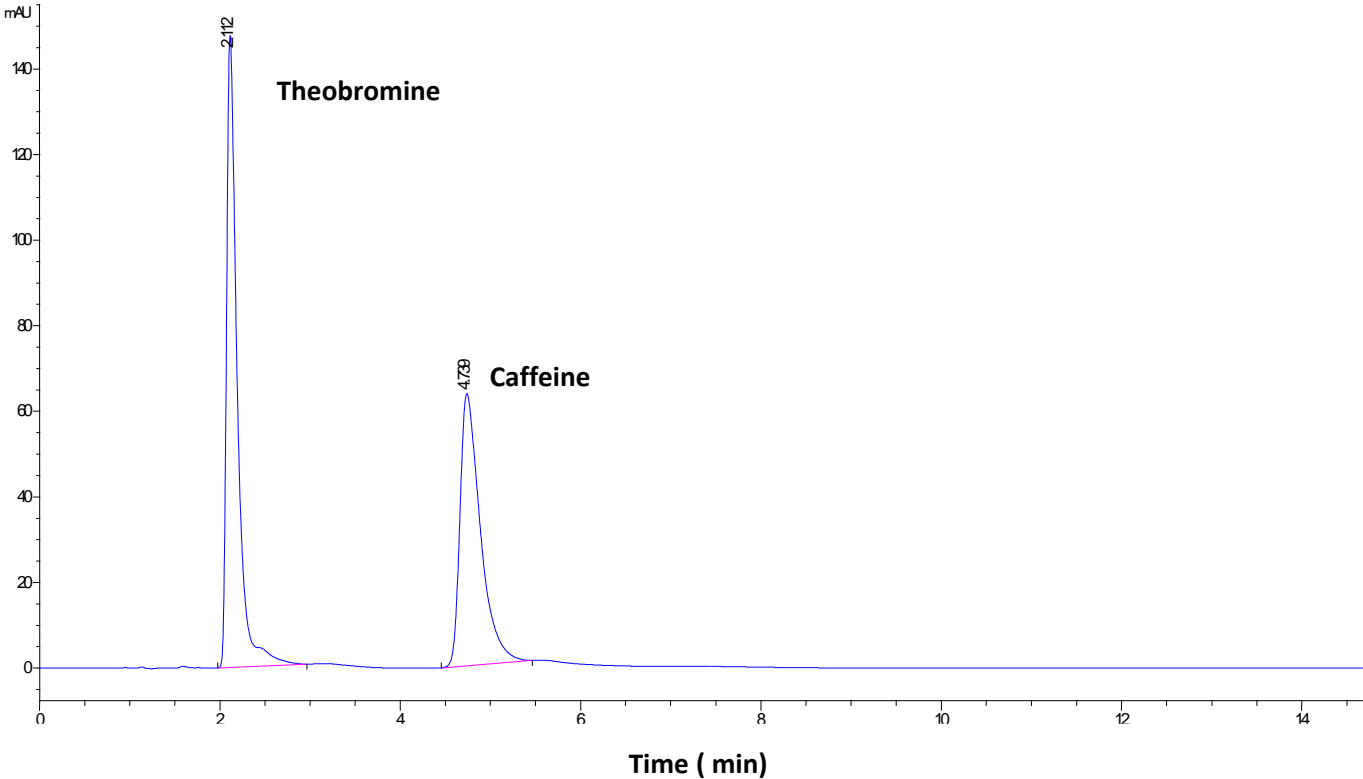

Supplement: Supplementary file 1 [file foods-12-02583-s001.zip › foods-2415752-supplementary.pdf]
